# SGLT2 Inhibitors as a Therapeutic Option for Both Acute and Chronic Refractory Hypomagnesemia in Diabetic and Nondiabetic Patients: A Multicenter Case Series

**DOI:** 10.1155/crin/5615339

**Published:** 2025-09-09

**Authors:** Fatima Ayub, Praveen Errabelli, Abedalrahman Almashayekh, Ahmed Abdallah, Md Rajibul Hasan, Manisha Singh, Nithin Karakala, Joseph Hunter Holthoff

**Affiliations:** ^1^Department of Nephrology, Central Arkansas Veterans Health System, Little Rock, Arkansas, USA; ^2^Department of Nephrology, University of Arkansas for Medical Sciences, Little Rock, Arkansas, USA; ^3^Department of Nephrology, Allina Health Foundation Minneapolis, Minneapolis, Minnesota, USA; ^4^Department of Internal Medicine, Unity Health–White County Medical Center, Searcy, Arkansas, USA

## Abstract

Sodium–glucose cotransporter 2 (SGLT2) inhibitors are widely used in patients with kidney disease and have been shown to increase serum magnesium levels. Case reports have described their role in correcting hypomagnesemia; however, there is limited evidence regarding their efficacy in patients with renal magnesium wasting. Furthermore, data regarding their role in acute treatment and sustained efficacy to treat hypomagnesemia are lacking. We present a multicenter retrospective, observational case series from two U.S. medical centers describing five patients with refractory hypomagnesemia who experienced significant improvement following initiation of SGLT2 inhibitors. Patients 1–4 showed marked renal magnesium wasting with severe symptomatic hypomagnesemia which responded robustly to SGLT2 inhibitor therapy. Even though Patient 5 did not have renal magnesium wasting, hypomagnesemia still improved with the addition of an SGLT2 inhibitor. Furthermore, the addition of an SGLT2 inhibitor acutely improved the hypomagnesemia of Patients 1 and 2 in an inpatient setting, and Patients 3–5 demonstrated sustained improvement of hypomagnesemia across extended outpatient follow-up. The improvement of hypomagnesemia was irrespective of the diabetic status of the patient. All cases resulted in substantial reduction or cessation of magnesium (Mg) supplementation. These findings suggest a novel therapeutic application of SGLT2 inhibitors for managing intractable hypomagnesemia, both acutely and chronically, regardless of the diabetes being the primary culprit.

## 1. Introduction

SGLT2 inhibitors were originally developed for optimizing glycemic control in Type 2 diabetes mellitus (T2DM) and have since gained recognition for their cardiorenal benefits in both diabetic and nondiabetic populations [1]. Beyond these effects, several smaller studies have noted a class effect of increasing serum magnesium levels, particularly in patients with T2DM [2–4]. Hypomagnesemia is common in diabetes, affecting 14%–48% of individuals compared to 2.5%–15% in nondiabetic populations [5–9]. While proposed mechanisms for this magnesium-sparing effect include improved insulin sensitivity and enhanced renal reabsorption via transient receptor potential Melastatin 6 (TRPM6) channels [3], the efficacy of SGLT2 inhibitors in nondiabetic patients with hypomagnesemia remains underexplored.

**Figure 1:**
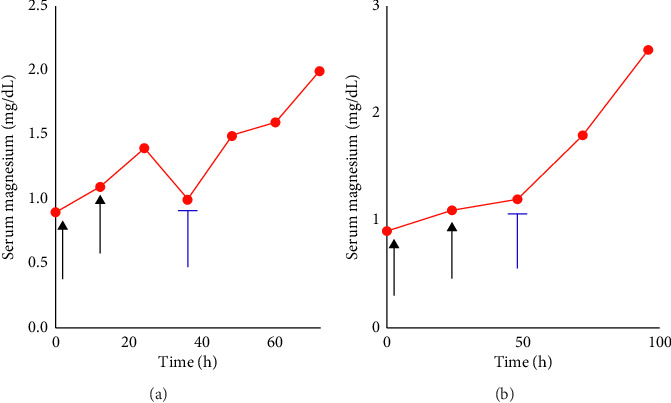
Acute effects of the treatment of hypomagnesemia with an SGLT2 inhibitor in the inpatient setting. Black arrows represent the administration of intravenous magnesium replacement, and blue brackets represent the initiation of SGLT2-I therapy. Patient #1 is displayed in panel (a) and Patient #2 is displayed in panel (b).

**Figure 2:**
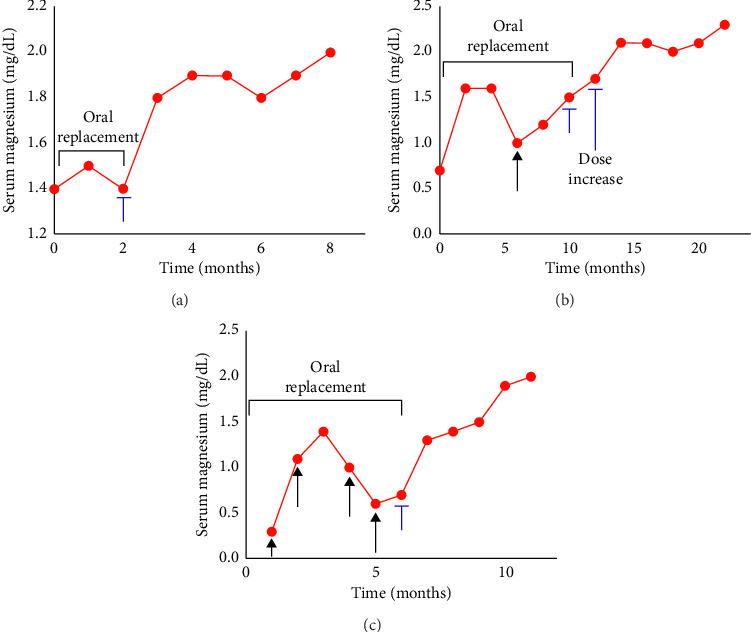
Chronic effects of the treatment of hypomagnesemia with an SGLT2 inhibitor in the outpatient setting. Black arrows represent the administration of intravenous magnesium replacement, and blue brackets represent the initiation of SGLT2-I therapy. Patient #3 is displayed in panel (a), Patient #4 is displayed in panel (b), and Patient #5 is displayed in panel (c).

Shah et al. reported four nondiabetic cases of hypomagnesemia successfully treated with SGLT2 inhibitors, suggesting a potential role for these agents in modulating renal magnesium handling beyond glycemic control [4]. Building on those findings, we present five cases (Table 1) of both diabetic and nondiabetic patients from two U.S. medical centers, all with refractory hypomagnesemia unresponsive to conventional supplementation (defined by persistent hypomagnesemia despite oral and/or intravenous replacement of magnesium for at least 2 weeks). Each case demonstrated sustained clinical and biochemical improvement following SGLT2 inhibitor therapy (Figures 1 and 2). Empagliflozin was initiated at the lowest available dose and subsequently increased if needed based on serum magnesium levels observed during follow-up visits.

These findings presented here expand the current understanding of SGLT2 inhibitors in the management of hypomagnesemia, while laying the foundation to pursue larger clinical investigation to establish a role for SGLT2 inhibition in the treatment of hypomagnesemia.

## 2. Case 1

A 67-year-old Caucasian female nondiabetic patient, recently diagnosed with Stage 1 triple-negative breast cancer, was transferred from an outside hospital (OSH) due to concerns for hemophagocytic lymphohistiocytosis (HLH). The patient had undergone several cycles of chemotherapy with adriamycin, cyclophosphamide, pembrolizumab, cisplatin, and paclitaxel for her breast cancer, with the last dose received 3 months prior to this presentation for generalized weakness and neutropenic fever at OSH. Her hospital course was complicated by COVID-19 infection, CMV viremia, and candidemia. She developed pancytopenia, initially thought to be secondary to HLH, but further workup at our hospital ruled out this possibility, and chemotherapy was determined to be the culprit. Throughout her hospitalization, she was found to have profound hypomagnesemia. Her initial serum magnesium was 0.9 mg/dL, which improved to 1.3-1.4 mg/dL with 10 doses of IV magnesium sulfate 4 g and oral magnesium oxide 800 mg twice daily supplementation. However, the levels continued to fluctuate and even declined despite ongoing oral replacement. Additionally, she could not tolerate magnesium oxide and developed intractable watery diarrhea, after which her magnesium oxide was stopped.

Nephrology was consulted for refractory hypomagnesemia. Her serum creatinine (Cr) and all other electrolytes were within normal limits, except for a serum potassium level of 3.4 mmol/L. At the time of initial consult, her diarrhea had resolved. We obtained a 24-h urinary magnesium excretion, which measured 79 mg over 24 h. Using the fractional excretion of magnesium (FEMg) formula, the FEMg was calculated using the formula (FEMg (%) = (*Urine Mg* × *Serum Cr*) ÷ (*Serum Mg* × *Urine Cr* × *0.7*) × 100, in which *0.7* accounts for the fraction of ultrafilterable serum magnesium). The result was 16.2%, indicating significant renal Mg wasting. Additionally, the sample was undercollected indicating that the urinary magnesium excretion was likely much higher. The patient was started on empagliflozin 10 mg, which led to significant improvement in her serum Mg levels to 2 mg/dL (Figure 1(a)). The patient was discharged but failed to follow up with the outpatient nephrology clinic.

## 3. Case 2

A 72-year-old frail woman with poorly controlled T2DM and a history of gastric bypass surgery presented to the emergency department with worsening fatigue and persistent hypomagnesemia. Over the preceding months, she had experienced generalized weakness, tremors, and reduced appetite. Her medical history was notable for chronic electrolyte disturbances, including hypomagnesemia, hypokalemia, hypocalcemia, and metabolic alkalosis.

Despite ongoing oral magnesium supplementation, her serum magnesium remained critically low. Home medications included metformin, insulin aspartate, and pantoprazole. On physical examination, she appeared frail, with resting tremors and signs of cognitive impairment. Her vital signs were stable. Laboratory tests revealed a serum Mg level of 0.9 mg/dL. Pantoprazole was discontinued and both oral magnesium oxide (400 mg every 6 h) and intravenous magnesium sulfate 2 g (for 6 doses) were initiated, resulting in a modest increase in serum magnesium to 1.2 mg/dL. However, her treatment course was complicated by magnesium oxide–induced diarrhea, which further impaired gastrointestinal absorption and limited the effectiveness of oral therapy.

A 24-h urine collection revealed urinary Mg excretion of 171 mg and a FEMg of 18%, confirming significant renal magnesium wasting. Genetic testing for Gitelman syndrome was negative.

Given the refractory nature of her hypomagnesemia and persistent symptoms, an SGLT2 inhibitor was initiated, resulting in significant clinical and biochemical improvement, with serum magnesium levels rising to 2.3 mg/dL (Figure 1(b)). The patient unfortunately passed away due to terminal illness, and hence, we did not have a long-term follow-up for her.

## 4. Case 3

A 58-year-old obese Caucasian woman with a history of T2DM, hypertension, long QT syndrome (status post permanent pacemaker), coronary artery disease (status post percutaneous coronary intervention), subtotal parathyroidectomy, and gastroesophageal reflux disease (GERD) on chronic proton pump inhibitors (PPI) was referred to the nephrology clinic for evaluation of chronic hypomagnesemia.

Contributing risk factors included poorly controlled diabetes, prolonged PPI use, and chronic diarrhea which had recently improved with loperamide. She had been on magnesium supplementation for over 12 years with serum magnesium levels persistently between 1.4 and 1.5 mg/dL. Despite oral supplementation, she experienced recurrent episodes of fatigue and muscle cramping, leading to frequent emergency department visits requiring intravenous magnesium infusions of 2–4 g.

Various oral magnesium formulations had been trialed but were poorly tolerated due to diarrhea. She was subsequently maintained on monthly IV magnesium sulfate infusions through an outpatient infusion center, which stabilized serum magnesium levels but failed to relieve her symptoms.

A 24-h urine magnesium collection was not performed, as the patient declined to discontinue supplementation due to concerns about triggering long QT-related arrhythmias. A random urine magnesium-to-creatinine ratio was 0.19 mg/mg, suggestive of renal magnesium wasting but not confirmatory.

Empagliflozin (10 mg daily) was initiated, resulting in a marked improvement in serum magnesium levels to 1.8 mg/dL at one month follow-up. At that time, oral and IV supplementation was discontinued. The patient also reported complete resolution of her fatigue and cramping. Her magnesium levels remained stable between 1.9 and 2.0 mg/dL during the monthly follow-ups for 6 months (Figure 2(a)).

## 5. Case 4

A 64-year-old man with uncontrolled T2DM, obstructive sleep apnea (on CPAP), coronary artery disease (status post triple-vessel coronary artery bypass grafting), aortic stenosis (status post valve replacement), and Stage II chronic kidney disease (eGFR ∼70 mL/min) was referred to the nephrology clinic for evaluation of severe, chronic, and symptomatic hypomagnesemia. His home medications included lisinopril, spironolactone, warfarin, metformin, and insulin.

Six months prior, his serum magnesium was critically low at 0.7 mg/dL, and his HbA1c was 11.2%. He reported debilitating fatigue and muscle cramps significantly impairing daily functioning. Initial treatment with oral magnesium oxide 500 mg three times daily caused diarrhea, prompting a switch to magnesium lactate, which was also poorly tolerated. He became reliant on weekly intravenous magnesium infusions, which modestly increased his serum magnesium to 1.5 mg/dL but did not normalize it or alleviate his symptoms. He was placed back on oral magnesium oxide with the reduced dose frequency of 500 mg twice daily with negligible improvement.

A 24-h urinary magnesium collection demonstrated a urinary excretion of 90 mg/day and a FEMg of 12%, confirming significant renal magnesium wasting. Empagliflozin (10 mg daily) was initiated. At 2-month follow-up, his serum magnesium improved to 1.5 mg/dL followed by an increase in the dose of empagliflozin to 25 mg daily. Over the subsequent months, his serum magnesium levels steadily improved, reaching 2.0 mg/dL and consistently remaining above 2.1 mg/dL. He no longer required intravenous magnesium supplementation, and his oral magnesium oxide was reduced to 500 mg once daily followed by discontinuation (Figure 2(b)). His symptoms of fatigue and cramping resolved completely.

## 6. Case 5

A 62-year-old man with a history of well-controlled T2DM, hypertension, coronary artery disease (status post percutaneous coronary intervention), and GERD (on chronic PPI) was referred to the nephrology clinic for management of chronic hypomagnesemia with baseline serum magnesium levels ranging from 0.7 to 1.1 mg/dL, despite receiving multiple oral magnesium preparations. His lowest documented serum Mg level was 0.3 mg/dL. He required frequent intravenous magnesium infusions, at least twice monthly, administered in the emergency department or infusion clinic. His hypomagnesemia persisted despite PPI discontinuation and the use of maximum tolerated doses of oral magnesium oxide and magnesium chloride. He reported intractable fatigue and recurrent leg cramps.

Initial nephrology evaluation included a 24-h urine collection, which revealed a urinary magnesium excretion of 47 mg/day and a FEMg of 2%, not consistent with renal magnesium wasting.

He was started on empagliflozin 10 mg daily, which was increased to 25 mg daily at 2-month follow-up. Over the following 6 months, his serum magnesium levels improved progressively—from 0.7 to 2 mg/dL, accompanied by tapering oral magnesium chloride 143 mg to twice daily and subsequently reduced to once daily (Figure 2(c)). His clinical symptoms, including fatigue and leg cramps, have resolved.

## 7. Discussion

Magnesium is a predominantly intracellular cation, and its homeostasis relies on a tightly regulated balance between gastrointestinal absorption and renal excretion. While conventional management typically includes discontinuing contributing medications and initiating magnesium supplementation, these strategies are frequently ineffective in patients with underlying renal magnesium wasting, warranting the utilization of novel approaches [1]. SGLT2 inhibitors, originally developed to optimize glycemic control, have increasingly been recognized for their role in modulating magnesium homeostasis.

Hypomagnesemia, defined as a serum magnesium concentration below 0.7 mmol/L, is particularly prevalent in individuals with T2DM with reported rates ranging from 13% to nearly 48%, significantly higher than the general population [5, 6]. This association was recognized as early as the mid-20th century and is largely attributed to insulin resistance impairing renal magnesium reabsorption resulting in increased urinary losses [1, 7, 8]. In patients with T2DM, renal magnesium handling is often compromised due to a combination of hyperglycemia, insulin resistance, and hyperinsulinemia [9]. Given this pathophysiologic basis, SGLT2 inhibitors may help restore magnesium balance not only by lowering plasma glucose levels but also by improving insulin sensitivity.

Approximately 15%–25% of the filtered magnesium is reabsorbed in the proximal tubule through passive paracellular transport, which is facilitated by an electrochemical gradient maintained by sodium reabsorption and the sodium–hydrogen exchanger 3 (NHE3) [10, 11]. Glomerular hyperfiltration driven by elevated plasma glucose enhances urinary flow and dilutes luminal magnesium concentrations, reducing the concentration gradient needed for effective passive reabsorption in the proximal tubule. This disruption triggers a self-perpetuating cycle of magnesium wasting [5].

In the thick ascending limb (TAL) of the loop of Henle, approximately 60%–70% of filtered magnesium is reabsorbed via claudin-16- and claudin-19-regulated paracellular channels. The driving force for this process is the lumen-positive transepithelial voltage generated by NKCC2-mediated sodium reabsorption [11, 12]. While it is well established that diabetes increases renal magnesium losses, the specific alterations in claudin-16 regulation remain under investigation. Experimental studies in mice suggest that insulin promotes magnesium reabsorption in the TAL; therefore, insulin resistance or deficiency in T2DM may diminish this stimulatory effect and exacerbate magnesium loss [13]. However, this hypothesis is speculative, and further studies are warranted.

The distal convoluted tubule (DCT), although responsible for only around 10% of magnesium reabsorption, plays a pivotal role in determining the final urinary magnesium concentration as no significant reabsorption occurs beyond this point [14]. Magnesium handling in the DCT is active and transcellular, primarily mediated by the TRPM6 channel. Studies in mice have shown a direct influence of epidermal growth factor (EGF), insulin, and pH on TRPM 6 function [15, 16]. Notably, TRPM6 has been identified as a key downstream target of insulin signaling, and its dysfunction has been implicated in the hypomagnesemia frequently observed in T2DM [5]. While insulin typically enhances TRPM6-mediated magnesium uptake, insulin resistance may reduce TRPM6 expression or activity impairing magnesium conservation [7]. Moreover, evidence suggests that in diabetes, sodium and magnesium reabsorption in the DCT may become uncoupled, a departure from the typical relationship seen in Gitelman syndrome or thiazide diuretic use in which decreased sodium reabsorption is associated with hypomagnesemia. This paradox highlights the need for further investigation into the insulin-resistant DCT phenotype and the regulatory crosstalk between sodium and magnesium transporters [5].

**Figure 3:**
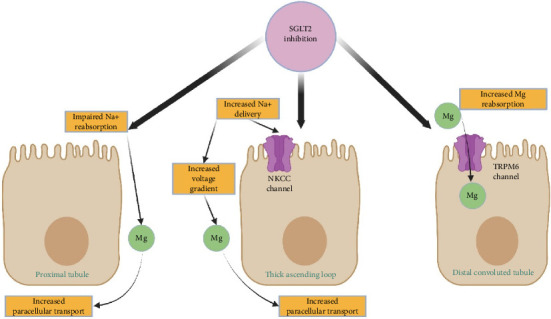
Proposed mechanism of an SGLT2 inhibitor to increase renal magnesium reabsorption.

Several mechanisms have been proposed to explain the magnesium-sparing effect of SGLT2 inhibitors (Figure 3). In the proximal tubule, SGLT2 blockade increases luminal electrical potential, enhancing paracellular magnesium reabsorption [4, 17]. SGLT2 blockage also increases the distal sodium delivery to TAL. In the TAL, augmented sodium delivery activates NKCC2 which strengthens the transepithelial voltage gradient to favor magnesium uptake [10]. In the DCT, emerging evidence suggests that SGLT2 inhibitors may enhance TRPM6-mediated magnesium transport, although the precise regulatory pathways are not yet fully defined [2, 4]. It is hypothesized that by improving insulin sensitivity, SGLT2 inhibitors may facilitate better insulin receptor activation, thereby promoting the trafficking and incorporation of TRPM6 channels into the apical membrane, helping to interrupt the cycle of insulin resistance and magnesium loss [5]. Another proposed mechanism by which SGLT2 inhibitors may support magnesium homeostasis involves glucagon-mediated stimulation of magnesium reabsorption. In experimental studies using mouse DCT cells, glucagon was shown to directly enhance Mg^2+^ uptake via a cAMP-dependent signaling pathway, like the effect observed with arginine vasopressin (AVP) [18]. Given that SGLT2 inhibition can lead to mild volume contraction and increased glucagon levels, both observed effects of this drug class, it is plausible that glucagon contributes to renal magnesium conservation in this setting [19]. Additionally, SGLT2 inhibition in pancreatic α-cells has been shown to influence intracellular pH and ionic flux, leading to increased glucagon secretion [18, 20]. Elevated glucagon levels may subsequently stimulate magnesium reabsorption by enhancing TRPM6 expression or activity in the DCT, thereby promoting active magnesium transport [18, 21]. While some studies suggest that this effect is mediated through SGLT1 in α-cells, others indicate that glucose lowering itself is a key driver of glucagon release during SGLT2 inhibitor therapy [22]. Importantly, individual variability in response to SGLT2 inhibitors may be partly attributed to differences in SGLT1 versus SGLT2 expression within pancreatic α-cells. Since SGLT1 is more consistently expressed in these cells, its differential activity could help explain why some patients experience greater glucagon-mediated magnesium retention than others [4, 21]. However, this proposed pathway also remains hypothetical, and further studies are needed to validate the role of glucagon in magnesium handling and its interaction with SGLT2 inhibitor therapy.

Although initially studied in diabetic populations, emerging evidence suggests that SGLT2 inhibitors may also benefit nondiabetic individuals with hypomagnesemia. A case series by Shah et al. and another by Ray et al. described four nondiabetic patients and three with diabetes who experienced marked improvement in serum magnesium levels following initiation of SGLT2 inhibitors, without changes in FEMg, suggesting nonrenal mechanisms, such as intestinal absorption [4, 8]. Magnesium absorption in the intestine occurs via two primary pathways: a passive paracellular route and an active transcellular mechanism. The passive pathway is driven by concentration gradients and occurs predominantly in the small intestine, accounting for the majority of magnesium uptake under normal dietary conditions. In contrast, the active transcellular pathway is mediated primarily by TRPM6 and TRPM7 channels, which are expressed in the apical membranes of enterocytes, particularly in the distal small intestine and colon. These channels play a critical role in maintaining magnesium balance, especially during periods of low dietary intake. Disruption of TRPM6 function has been implicated in various hereditary and acquired hypomagnesemic disorders. Notably, chronic PPI use has been associated with impaired intestinal magnesium absorption, likely due to reduced expression of active transporters and changes in luminal pH, underscoring the importance of understanding these absorptive mechanisms [14, 16, 23].

In contrast to traditional SGLT2 inhibitors, sotagliflozin, a dual SGLT1/SGLT2 inhibitor, has demonstrated the potential to enhance gastrointestinal magnesium absorption, likely through SGLT1-mediated mechanisms. Shah reported a nondiabetic patient with refractory hypomagnesemia who experienced a sustained rise in serum magnesium levels after starting sotagliflozin, without corresponding increases in urinary magnesium excretion, suggesting that the improvement was primarily due to increased intestinal uptake rather than renal conservation [24]. This observation provides a useful comparator to Case 5 in our study, in which the patient had PPI-induced hypomagnesemia and was treated with empagliflozin, a selective SGLT2 inhibitor with minimal SGLT1 inhibition. Given that empagliflozin primarily affects renal glucose handling and has limited intestinal SGLT1 activity, its benefit in cases of impaired magnesium absorption, such as PPI-induced hypomagnesemia, may be modest. These contrasting outcomes underscore the need for controlled trials to better delineate the role of SGLT1 inhibition in magnesium homeostasis, especially in nondiabetic and gastrointestinally driven hypomagnesemic states.

Other hypotheses for the ability of SGLT2 inhibitors to increase serum Mg levels outside of renal reabsorption have also been proposed (Table 2). One proposed mechanism is vasopressin-mediated enhancement of magnesium reabsorption [18]. Specifically, the osmotic diuresis induced by SGLT2 inhibition can lead to increased plasma osmolality, which stimulates vasopressin release [25]. In turn, AVP has been shown to directly stimulate Mg uptake in mouse DCT cells via a cAMP-dependent pathway [18].

The patient in Case #1, a nondiabetic patient with cisplatin-induced renal magnesium wasting [26–28], exhibited substantial biochemical improvement after initiation of empagliflozin, highlighting a potential role for SGLT2 inhibition even in the absence of diabetes. These findings reinforce the possibility that SGLT2 inhibitors may exert magnesium-sparing effects via nonglycemic pathways that merit further mechanistic investigation. Cases #2–4 involved diabetic patients with refractory hypomagnesemia and evidence of renal magnesium wasting. All responded favorably to SGLT2 inhibition, supporting its role in correcting magnesium deficits likely driven by insulin resistance and impaired renal tubular reabsorption. Although Case #5 involved a well-controlled diabetic patient, the primary etiology of hypomagnesemia was suspected to be chronic PPI use, which introduces a potential confounding factor. PPI-induced hypomagnesemia is widely regarded as a class effect and is believed to impair intestinal magnesium absorption, rather than renal reabsorption, as reflected by a low FEMg in affected individuals [23, 29]. These findings reinforce those of others and suggest that the mechanisms of SGLT2 inhibition to increase serum Mg levels may be both renal and extrarenal-driven.

## 8. Conclusion

To our knowledge, this is the first multicenter case series to report the successful use of SGLT2 inhibitors in correcting refractory hypomagnesemia in both diabetic and nondiabetic individuals. Additionally, we present evidence supporting the use of SGLT2 inhibition as a treatment for hypomagnesemia in the acute setting. The observed normalization of serum magnesium levels and reduced reliance on supplementation underscore the potential of SGLT2 inhibitors as a novel therapeutic strategy for managing renal magnesium wasting. However, these findings should be interpreted in the context of several limitations, including the small sample size, retrospective and observational design, limited long-term follow-up for the initial cases, the presence of potential confounders, and the absence of a control group. Further investigation, particularly through prospective, randomized controlled trials and laboratory-based studies, is essential to validate these findings and to elucidate the underlying mechanisms by which SGLT2 inhibitors modulate magnesium homeostasis across diverse patient populations.

## Figures and Tables

**Table 1:** Summary of baseline characteristics, metabolic profile, and changes observed in patients treated with SGLT2 inhibitors.

Case no	Sex, age	Etiology of hypomagnesemia	Baseline Mg (mg/dL)	Baseline eGFR (mL/min/1.73 m^2)	Baseline Hba1c (%)	Supplementation pre-empagliflozin	FEMg (%)	Post-treatment Mg (mg/dL)	Change in Mg supplementation
1	F, 67	Platinum-based chemotherapy	0.9	> 90	N/A	Oral and IV magnesium oxide	16.2	2	Discontinued oral and IV magnesium replacement
2	F, 72	GI malabsorption, PPI, T2DM	0.9	> 90	9.1	IV and oral magnesium oxide	18	2.3	Discontinued, oral and IV magnesium replacement
3	F, 58	Chronic diarrhea, T2DM, PPI	1.4	> 90	6.8	Monthly IV magnesium sulfate	N/A	2	IV magnesium discontinued
4	M, 64	Diabetic renal magnesium wasting	0.7	> 90	7.9	Oral magnesium oxide and magnesium lactate, IV magnesium	12	2	IV and oral magnesium discontinued
5	M, 62	PPI	0.3	> 90	6.1	Oral magnesium oxide and magnesium chloride, IV magnesium	2	2.1	IV magnesium discontinued, oral magnesium reduced and later discontinued

**Table 2:** Proposed mechanisms of magnesium reabsorption enhancement by SGLT2 inhibitors in nondiabetic patients.

Proposed mechanism	Segment/target	Effect	Likely role in nondiabetics?
Vasopressin secretion 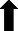	Collecting duct	Enhances Mg^2+^ reabsorption	Yes

Glucagon secretion 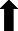 via α-cell pH shift	DCT (TRPM6)	Stimulates Mg^2+^ transport	Yes
SGLT 1/2 expression in α-cells	Pancreas 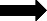 kidney	Modulates hormonal control of renal Mg^2+^ handling	Possibly

## Data Availability

The data that support the findings of this study are available on request from the corresponding author. The data are not publicly available due to privacy or ethical restrictions.
